# Does time from diagnostic CT until surgical evacuation affect outcome in patients with chronic subdural hematoma?

**DOI:** 10.1007/s00701-018-3620-y

**Published:** 2018-07-24

**Authors:** Shaian Zolfaghari, Nils Ståhl, Henrietta Nittby Redebrandt

**Affiliations:** 1 The Rausing Laboratory, Division of Neurosurgery, Department of Clinical Sciences Lund, Lund University, 221 85 Lund, Sweden; 2 Department of Neurosurgery, Skåne University Hospital, Lund, Sweden

**Keywords:** Chronic subdural hematoma, Time, Outcome, GCS

## Abstract

**Background:**

Chronic subdural hematoma (CSDH) is one of the most common neurosurgical conditions. Patients diagnosed with CSDH’s are often planned for subacute surgery. This means that time from diagnostic CT scan until actual surgery might often be prolonged. There are no previous studies that highlight the effect of delayed intervention in this population.

**Method:**

Patients that underwent surgical evacuation for a CSDH at Skåne University Hospital between 1 January 2015 and 31 December 2016 were included in this retrospective cohort study (*n* = 179). The primary aim was to determine if time from initial diagnosis by head-CT until surgical evacuation had a significant effect on outcome. The following was assessed by mortality, re-operation, number of days spent in hospital, discharge to home/institution, and functional outcome assessed by GOS. Secondary aims were to evaluate the effect of NOAC, vitamin K antagonists, and antiplatelet drugs on time from CT to surgery and re-operation frequency.

**Results:**

Mean time from diagnostic CT scan until surgery was 76 h. No significant relationship was found between time from CT to surgical evacuation and number of days spent in hospital, discharge to own home/institution, 1-year mortality, or outcome assessed by GOS at discharge from hospital. The clear majority (95.5%) of the patients were GCS ≥ 13 pre-operatively. No correlation could be seen between use of NOAC, vitamin K antagonists, or antiplatelet drugs regarding the risk for reoperation within 6 months, and no correlation between the use of these agents and time from CT to surgery. The 30-day mortality was too low to draw any statistically significant conclusions (*n* = 4).

**Conclusion:**

In this retrospective cohort study, we could conclude that a delay from initial diagnosis confirming a CSDH to surgical evacuation had no negative effect on outcome when surgery was performed within the time frames and on patients with pre-operatively favorable GCS scores (≥ 13) outlined in our study.

## Introduction

Surgical evacuation of chronic subdural hematomas (CSDH) is one of the most common neurosurgical procedures. The incidence of CSDH is approximated between 8.2 to 14 persons per 100,000/year showing a trend towards an increase in the incidence of CSDH in the coming decades [1, 2]. CSDHs have several established predisposing risk factors such as advanced age, previous trauma to the head, anticoagulant and antiplatelet medication, and history of alcohol abuse contributing to global cerebral atrophy [1, 3].

The presenting symptoms of a CSDH can vary and take on several different appearances. Most commonly, patients present with a high Glasgow coma scale (GCS) score together with gait disturbance and falls, limb weakness, headaches, mental deterioration, or acute confusion [4]. CSDH’s can often mimic other common neurological pathologies.

Regarding acute subdural hematomas (ASDHs), studies have indicated that surgical evacuation is urgent, and a 4-h rule has been presented. Patients operated within 4 h of injury had a mortality rate of 30%, as compared to 90% mortality if surgical evacuation was delayed more than 4 h [5]. In the case of a chronic subdural hematoma, the patient usually has a slow development of an intracranial mass lesion. Thus, there is rarely the need for an extremely urgent operation, and patients might have to wait several days for different reasons such as logistical reasons, anticoagulants, and antiplatelet drugs which must be reversed.

In this retrospective cohort study, our primary aim is to investigate if time from diagnostic CT scan until definitive treatment with surgical evacuation affects outcome in patients with unilateral CSDH. Outcome was assessed by GOS at discharge, days spent in hospital, mortality, re-operation, and discharge to home/institution. The secondary aims consist of investigating the effects of anticoagulants on reoperation frequency and whether the use of these drugs increases time from CT to surgery.

## Methods and materials

### Patient population

Patients over 18 years of age and with ICD code AAD10 that underwent surgical evacuation of unilateral CSDH between 1 January 2015 and 31 December 2016 at the Department of Neurosurgery, Skåne University Hospital, and were living in Scania County (the most southern part of Sweden) were eligible for this retrospective longitudinal cohort study. Exclusion criteria were patients diagnosed with bilateral CSDHs, simultaneous intracranial hemorrhages, and patients with cerebral shunts. To be able to follow up all the included patients after transfer from our clinic, we included the patients who live in Scania, and not the other southern counties who also refer patients to our clinic. A total of 380 patients were operated for CSDH during the above listed period, including patients living in other parts of Sweden. A total of 179 patients met the requirements for inclusion in the study.

### Surgical procedure

At the Department of Neurosurgery in Lund, patients are mainly operated with a mini-craniotomy where three burr holes are placed immediately adjacent to one another and connected. For most patients, local anesthesia is used. The subdural hematoma is irrigated with Ringer Acetate using a soft catheter until there is no residual exchange of blood in the fluid. This is followed by insertion of a passive subdural drainage for 24 h and bed rest. After 24 h, the drainage is removed, and the patients are mobilized.

### Study variables

All data was retrieved using the hospital’s electronic charting system.

A classification and regression tree based upon the analysis of 986 patients who were operated due to chronic subdural hematomas could predict outcome based on neurological status on admission, age, brain atrophy, thickness and density of hematoma, antiplatelet, and anticoagulant therapy [6]. Since brain atrophy can be difficult to measure, we chose to use previously diagnosed dementia as a marker instead [6]. Previous diagnosis of either Alzheimer’s disease, vascular dementia, Lewy body dementia, or frontotemporal dementia in our cohort was registered as dementia. We did not register subdural accumulation of air, which was a prognostic factor in the aforementioned study, since our patients do not undergo post-operative CT on a regular basis. Pre-operative status was assessed by hemiparesis, aphasia/dysphasia, GCS grade, and Markwalder grading score (see Table 1). Markwalder score was assessed by analyzing notes describing patient’s pre-operative status. Pre-operative head-CT characteristics in a form of diameter, midline shift, and attenuation of the hematoma (hypodense, isodense, and hyperdense) were registered. The maximal diameter of the hematoma in the coronal plane was registered. Pre-operative head-CT characteristics for CSDH were registered from the head-CT closest in time to surgical evacuation. In addition, we registered comorbidities such as ischemic heart disease, pulmonary disease, coagulopathies, and longtime abuse of alcohol.

**Table 1:** The five grades of the Markwalder scale

Markwalder scale	(Markwalder et al. 1981)
Grade 0	Absence of neurological symptoms or deficits
Grade 1	Patient alert and oriented; mild symptoms, such as headache or nausea; absent or mild symptoms or neurological deficit, such as reflex asymmetry
Grade 2	Patient drowsy or disoriented with variable neurological deficits, such as hemiparesis
Grade 3	Patient stuporous but responding appropriately to noxious stimuli; several focal signs, such as hemiparesis
Grade 4	Grade 4: Patient comatose with absent motor response to painful stimuli; decerebrate or decorticate posturing

Documented trauma to the head in relation to the patient’s symptoms was registered. The patient’s state of independence was assessed in three categories consisting of complete independence in daily activities, living with assistance at home or living in a residential living facility. Time from head CT to surgical evacuation was documented. Time from CT to surgical evacuation was defined as total amount of hours from diagnostic CT from which the decision to perform surgery was made until actual surgical evacuation of the hematoma took place.

Characteristics of the surgical evacuation in a form of craniotomy or mini-craniotomy were registered. Re-operation within 6 months was registered. Major complications related to the standard of care given for the patients’ subdural hematoma were defined as seizures, post-operative hematomas, and misplacement of post-operative drain.

Outcome was registered in a form of Glasgow outcome scale (GOS) (Table 2), 1-month mortality, 3-month mortality, 1-year mortality, days spent in hospital (defined from date of admittance to discharge including stay on non-neurosurgical wards), and if the patient was discharged to home or assisted living facility. GOS was assessed by analyzing standardized notes in the patient’s electronic chart describing functional status at time of discharge.

**Table 2:** Depicting each of the GOS scores with definitions

Glascow outcome scale	(Jennett et al. 1975)
Score 1	Death—severe injury or death without recovery of consciousness
Score 2	Persistent vegetative state—severe damage with prolonged state of unresponsiveness and a lack of higher mental functions
Score 3	Severe disability—severe injuries with permanent need for help with daily living
Score 4	Moderate disability—no need for assistance in everyday life, employment is possible but may require special equipment
Score 5	Low disability—light damage with minor neurological and psychological deficits

### Statistical analysis

Data was collected and stored in Microsoft Excel (Microsoft Corporation) and exported to SPSS (IBM Corporation) for statistical analysis. All tables were constructed in Microsoft Word (Microsoft Corporation). For statistical comparison tests, a *p* value of < 0.05 was considered to be statistically significant. Descriptive statistics were calculated in Microsoft Excel. Time from CT to surgery and its correlation to GOS was analyzed with one-way ANOVA for ascertaining significant differences in mean values between the various GOS subgroups. Fisher’s exact test and independent samples *t* test was used to analyze the effect of time from CT to surgery, and correlations were analyzed with Pearson test. A *p* value < 0.05 was considered to be statistically significant.

## Results

### Patient characteristics

A total of 179 patients were included in the study, see Tables 3 and 4 for characteristics and surgical outcome. The 95.5% of the patients were GCS ≥ 13 pre-operatively, whereas the remaining 4.5% of the patients were GCS 6–12 pre-operatively.

**Table 3:** Study cohort characteristics, pre-operative data on general characteristics, ongoing medication, and comorbidities

Study cohort	Total, *n* = 179
Age (years) (mean ± SD)	73 ± 12
Independent living	86%
Dementia	8.3%
Antiplatelet (Trombyl, Clopidogrel)	24%
NOAC	4.4%
Vitamin K antagonist (Waran)	17%
NSAID	7.2%
Ischemic heart disease	22%
Alcohol abuse	7.8%

**Table 4:** Study cohort characteristics, perioperative information concerning pre-operative status, surgical evacuation, and outcome

Symptoms related to CSDH	Total, *n* = 179
Previously known trauma	59%
Pre-operative GCS	14 ± 1
Hemiparesis	25%
Dysphasia/aphasia	18%
Pre-operative Markwalder score	0.92 ± 0.70
Surgery with minicraniotomy	97.8%
Surgery with craniotomy	2.2%
Reoperation within 6 months	16.8%
Major post-operative complication	6.1%
GOS at discharge from hospital (mean ± SD)	4.4 ± 0.8
1-year mortality	12.2%

Pre-operative CT scan showed CSDHs with maximal diameter of 22 ± 6 mm (mean value) and 9.3 ± 4.3 mm midline shift (mean value). The patients spent on average 12.7 ± 13.6 days in hospital for treatment of CSDH. One-year mortality for the whole cohort (*n* = 179) was 12.2%. Severe outcome, graded GOS 1 to 3, was recorded in a total of 21 patients in our study cohort (GOS 1 *n* = 4; GOS 2 *n* = 0; GOS 3 *n* = 17).

The frequency of re-operation was 16.8% (30 patients). All the patients were re-operated on the same side within 6 months from the first surgical evacuation of the primary CSDH. There was no statistically significant correlation between reoperation and the use of anticoagulants and antiplatelet medication (NSAID, vitamin K antagonist, NOAC, or antiplatelet agents (Fisher’s exact test, non-significant)). Furthermore, there was no correlation between dementia and reoperations.

### Time from diagnostic CT scanning until surgical procedure

**Fig. 1:**
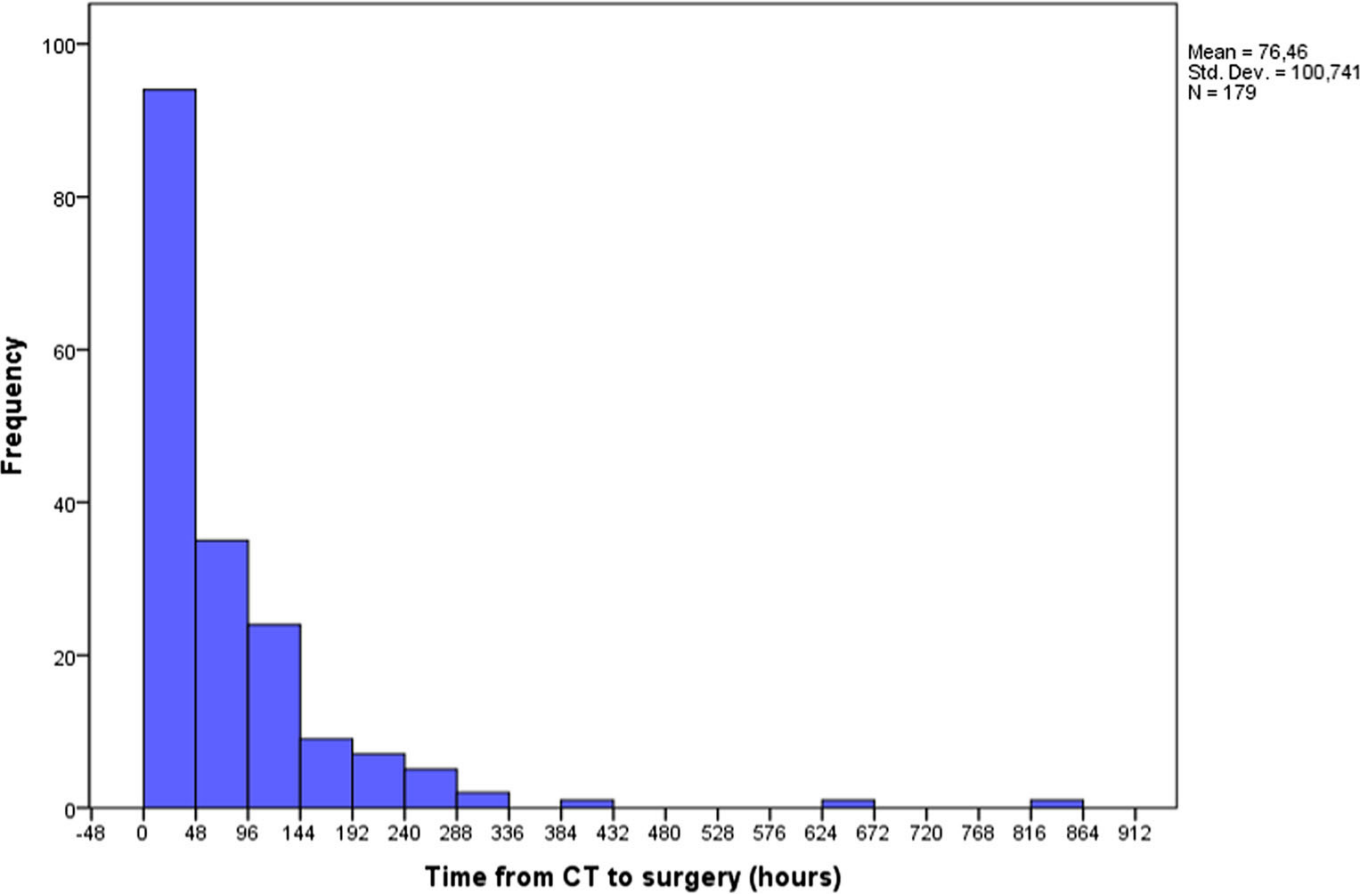
Bar graph depicting relationship between time from CT to surgery in relation to frequency (179 patients). Mean time from CT to surgery was 76.46 h with a SD of 100.74 h. Sixty patients were operated within 0 to 24 h, 36 patients between 24 and 48 h, 34 patients between 48 and 96 h, and 49 patients which were operated at 96 h and more from initial diagnostic CT

Mean time between diagnostic CT scanning upon which surgical decision was made until the actual surgical procedure took place was 76 h (Fig. 1). Seventy-three percent of the patients were operated within 96 h from diagnostic CT upon which the decision to operate was made. There was no correlation between time from CT until surgery and pre-operative GCS (Pearson correlation coefficient − 0.06). No correlation was found between the presence of paresis (Pearson correlation coefficient − 0.03) or aphasia (Pearson correlation coefficient − 0.02) and time from diagnostic CT scan until surgery.

**Fig. 2:**
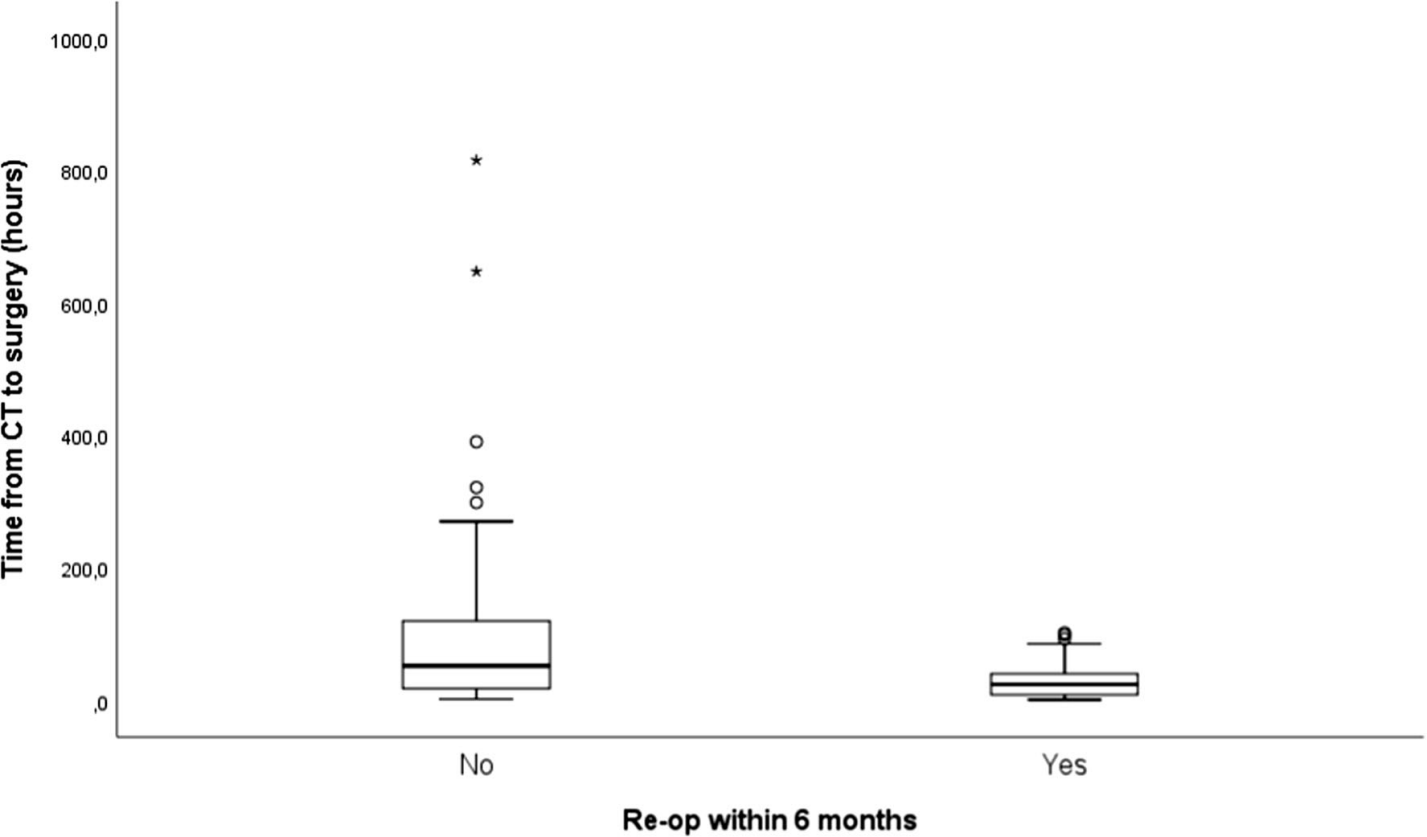
Figure depicting the mean time from diagnosis of CSDH by head-CT to surgical evacuation in two subgroups consisting of patients which did or did not undergo reoperation within 6 months of surgical evacuation of primary CSDH. There was an increased risk to undergo re-operation within 6 months when time from diagnostic head-CT until surgical evacuation was reduced (*p* = 0.007, independent samples *t* test)

There was no correlation between time from CT to surgery and discharge to home or not (Pearson correlation − 0.03), and likewise, no statistically significant correlation between time from CT to surgery and days spent in hospital (Pearson correlation 0.16). There was no correlation between time from CT to surgery and the 1-year mortality for our study cohort (Pearson correlation − 0.02). The use of any thrombocyte inhibitors and vitamin K antagonists prior to diagnosis did not significantly increase the time from CT to surgery (*p* = 0.093, independent samples *t* test). There was an increased risk to undergo re-operation within 6 months when time from diagnostic CT scans until surgery was reduced (*p* = 0.007, independent samples *t* test) (Fig. 2).

Patients were divided into the natural subgroups described by GOS protocol and analyzed in relation to time from diagnostic CT scan to surgical evacuation with one-way ANOVA. No significant relationship was found (*p* = 1.000). Time from CT to surgery was furthermore divided into four subgroups: 0–24, 24–48, 48–96, and 96+ h. Sixty patients were operated within 0 to 24 h, 36 patients between 24 and 48 h, 34 patients between 48 and 96 h, and 49 patients waited over 96 h from initial diagnostic CT scan until surgery took place. The new distribution was compared to the GOS score with Fisher’s exact test. No significant relationship was found (*p* = 0.409). Furthermore, there was no significant difference between preoperative GCS and time from CT until surgery (Pearson correlation − 0.077).

Forty-nine patients were surgically evacuated after 96+ h. The average time from diagnostic CT to surgical evacuation was 188.7 ± 131.2 h. We found that 36% of the patients had received antiplatelet medication and 22% of the patients had received a vitamin K antagonist. Approximately 86% of the patients were living independently before the presentation of the CSDH in this specific subgroup of patients. Patients that were surgically evacuated after 96+ h had an average delay of 57 h due to metabolization or reversal of anticoagulant or antiplatelet agents. This number is higher compared to the rest of the study cohort that averaged 25 h. Eighty-four percent were discharged to their homes. Outcome characteristics showed an average of GOS score 4.26 and a reoperation frequency of 4%. One-year mortality for patients surgically evacuated after 96 h was 14%.

### Dementia in relation to CSDH

We noted in our analysis that rather than time from CT until surgery, previously diagnosed dementia seemed to be an important factor. In this group, 1-year mortality was 40%. Average GOS at discharge was 3.5. Post hoc analysis revealed that patients with dementia had a significantly greater risk of being discharged in a state of severe/moderate disability according to GOS (*p* = 0.002, Fishers exact test).

## Discussion

In this retrospective cohort study, we were not able to identify any significant negative effect upon outcome correlated to time from diagnostic CT scan until surgery. On the contrary, it seems like the risk for re-operation within 6 months was reduced. Theoretically, it could be hypothesized that the use of antiplatelet medication/anticoagulants did not significantly increase the time from CT to surgery. This could be a possible explanation. But, on the other hand, no significant correlation could be found between the use of antiplatelet medication/anticoagulants and re-operation within 6 months. One major advantage with this study is that the patients are selected from a cohort, and that all patients from Scania who met the inclusion criteria could be included, and that follow up is available for all of them.

One possible confounding factor in this study could be that patients with more severe symptoms underwent surgery more urgently. However, we could see no correlation between preoperative GCS and delay until surgery. Worth having in mind here is that the patients in this cohort were in a relatively good condition, with a mean preoperative GCS 14 with only eight patients graded between GCS 3–12, meaning that statistical analysis of patients graded GCS 3–12 would be rather impractical to draw conclusions from. The correlations described in this study are thus applicable for surgical candidates with GCS ≥ 13.

Another possible confounding factor could be that it took time from the CT scan until the treating physician realized that the patient was a possible neurosurgical candidate. On the other hand, radiologists must alert the referring doctor if they find some major intracranial process on a CT scan, meaning that this confounding factor at least seems to be reduced.

The result from our study gives us some confidence that we do not harm our patients when we perform surgical evacuations of CSDH’s within a subacute timeframe, given that we perform the surgical evacuation within the timeframes presented in this study. Since this is a retrospective study, it has its limitations. However, out of an ethical point of view, it is difficult to perform a randomized prospective study where patients are randomized to a postponed surgical evacuation, while the neurosurgical service could theoretically be ready to operate on a clear subacute indication.

Post hoc analysis showed that dementia is a prognostically negative factor for patients who underwent surgery for CSDH. Our study showed that patients previously diagnosed with dementia were more likely to be discharged in moderate/severe disability (scored by GOS). Several studies have also identified dementia as a comorbidity that has a significant effect on patients being operated for CSDH. This could be primarily explained by a delay of diagnosis mediated by several reasons. Advanced dementia could imitate and mask the symptoms of CSDH for a longer period, but also due to cognitive impairment not allow the patient to report minor trauma or perceived symptoms. Patients diagnosed with dementia are also more dependent on family members and caregivers, thus making them less independent in their ADL [7, 8]. Also, dementia is more often associated with brain atrophy, further increasing the risk re-operation [6].

In our study, we had a 16.8% re-operation frequency and all the re-operations occurred within 6 months from primary CSDH evacuation and on the same side. Our numbers fall within the previously reported numbers of recurring CSDH’s of approximately 10–20% but are within the higher margin [2, 9]. The use of anticoagulants and antiplatelet medication prior to the first operation did not increase the risk for a re-operation. A study comparing different surgical techniques for evacuation of CSDH is ongoing.

A factor to take into consideration in future studies is the increasing use of NOAC, since cardiologists and neurologists report positive preventive effects of these agents [10]. The trend currently favors an increase in prescription of NOAC’s and a decrease in prescription of vitamin K antagonists such as Warfarin [11]. Currently, there is only one NOAC available with an antidote, Dabigatran® with the antidote Idarucizumab®. The remaining NOAC’s do not have an antidote available. The current recommendations for management of bleeding complications regarding NOAC state that patients needing to undergo urgent surgery need at least 12 h and ideally 24 h from last taken dose to allow for sufficient reversal of effect. In cases of patients with impaired renal function, more time is necessary to allow for sufficient coagulation (up to 48 h). The increasing use of NOAC might further increase time from diagnostic CT scan until surgery, since reversal of NOAC is more complicated as compared to vitamin K antagonists [12].

Limitations to this study are inherent to its retrospective design. The patients included in this study were all managed and treated by different neurosurgeons with no randomization of patients into each specific time interval. However, we believe that each neurosurgeon treated the patients by the same management principals employed at our institution. Randomized studies would eliminate selection bias, but ethical considerations need to be made carefully and might actually render a prospective study analyzing effects of time from CT to surgery difficult to perform. Thus, highlighting retrospective cohort analyses of this sort are of significant value.

## Conclusions

In this retrospective cohort study, we found that the time from diagnostic head-CT until surgical evacuation of CSDH had no negative effect on outcome using the values registered in this present study, when the patients were operated within the time frames presented herein and GCS ≥ 13 pre-operatively. Further research is planned in order to compare surgical methods and effects upon re-operation. Interestingly, it seemed like the risk for re-operation was increased when there was a shorter time frame from CT until surgery, rather than the contrary. Furthermore, a retrospective study of course has inherent limitations and to further evaluate the question a prospective observational study might be of value.
